# Accidental retropharyngeal dissection extending close to the right common carotid artery during nasotracheal intubation: a case report

**DOI:** 10.1186/s40981-023-00603-1

**Published:** 2023-02-28

**Authors:** Aki Okamoto, Yoshitaka Kawaraguchi, Masahide Fujita, Yasunobu Goto, Mitsuru Shimokawa

**Affiliations:** 1 Department of Anesthesiology, Nara City Hospital, 1-50-1 Higashikidera-cho, Nara-shi, Nara, 635-0833 Japan; 2 Department of Intensive Care, Nara City Hospital, 1-50-1 Higashikidera-cho, Nara-shi, Nara, 635-0833 Japan

**Keywords:** Nasotracheal intubation, Complication, Retropharyngeal dissection, Common carotid artery

## Abstract

**Background:**

Retropharyngeal dissection is a possible complication during nasotracheal intubation. We report a case of a retropharyngeal dissection extending close to the right common carotid artery occurring while inserting a nasotracheal tube.

**Case presentation:**

An 81-year-old woman, scheduled for laparoscopic and endoscopic cooperative surgery for a duodenal tumor under general anesthesia, sustained submucosal dissection of the retropharyngeal space during nasotracheal intubation. Postoperative computed tomography revealed retropharyngeal tissue injury extending close to the right common carotid artery. The patient was treated with prophylactic antibiotic therapy and discharged uneventfully on postoperative day 13.

**Conclusions:**

Submucosal dissection of the retropharyngeal tissue during nasotracheal intubation has a potential risk of major cervical vessel injury. Therefore, when the tip of the tube cannot be visualized within the oropharynx, clinicians must proceed with caution regarding the expected depth of the tube.

**Supplementary Information:**

The online version contains supplementary material available at 10.1186/s40981-023-00603-1.

## Background

Oral and maxillofacial surgery usually requires nasotracheal intubation to achieve a better surgical field. However, various complications, such as epistaxis [1], turbinectomy [2], or retropharyngeal dissection [3–5], have been reported to occur in association with resistance to the blind advancement of a tracheal tube inserted into the oropharynx via a nostril from the nasal cavity. However, to our knowledge, no reports have described the potential risk of cervical vessel injury caused by nasotracheal intubation. Herein, we report a case of a retropharyngeal dissection extending close to the right common carotid artery caused by a nasotracheal tube.

## Case presentation

An 81-year-old female (height = 1.48 m, weight = 48 kg) was scheduled for laparoscopic and endoscopic cooperative surgery for a duodenal tumor under general anesthesia. She had no relevant medical history. Nasotracheal intubation was requested by the endoscopist to avoid accidental extubation during the endoscopic maneuver. General anesthesia was induced with propofol 30 mg, remifentanil 0.6 mg, and rocuronium 40 mg intravenously; anesthesia was maintained with sevoflurane in oxygen and air. Before intubation, we cleaned the right nostril with cotton swabs soaked in 4% lidocaine and povidone-iodine. After applying a lubricating jelly to the right nasal passage, the lubricated Parker Flex-Tip nasal endotracheal tube (6.5 mm; Parker Medical, USA) was inserted blindly into the floor of the nose and then advanced by rotating clockwise. The depth of the tube was already 17 cm, so we tried to find the tube in the oropharynx by direct laryngoscopy. However, the tip of the tube could not be visualized; instead, the right submandibular subcutaneous tissue was observed to be bulging. We suspected a submucosal retropharyngeal dissection, and the tube was immediately removed. Brief mask ventilation was performed until conditions were suitable for intubation.

In a second attempt, a nasogastric tube (14 Fr, NIPRO, Japan) was inserted through the left nostril, and the tip of the tube in the oropharynx was confirmed by direct laryngoscopy. Then, the nasotracheal tube was put over it, and the tip of the endotracheal tube was also confirmed under direct laryngoscopy at the depth of 12 cm. After removing the nasogastric tube, nasotracheal intubation was performed successfully, and the tube was finally fixed at 29 cm on the nostril. Since there was no active bleeding in the pharynx or palpable cervical swelling, we decided to proceed with the surgery as planned. All vital signs were stable throughout the surgery. After the operation, we examined the pharynx again by direct laryngoscopy, and there were no remarkable findings. The nasotracheal tube was gently extubated in the operating room, and the patient was able to breathe normally. Considering the risk of airway obstruction caused by delayed retropharyngeal hematoma, the patient was transferred to the intensive care unit.

**Fig. 1:**
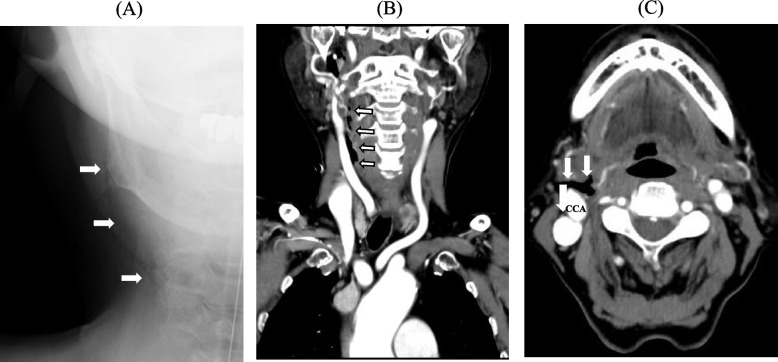
Postoperative chest X-ray (**A**) and contrast-enhanced computed tomography (**B**) (**C**) images. Subcutaneous emphysema was observed in the right side of the neck (**A**), and it extended up to the right carotid artery (**B**) (**C**). CCA, common carotid artery

**Fig. 2:**
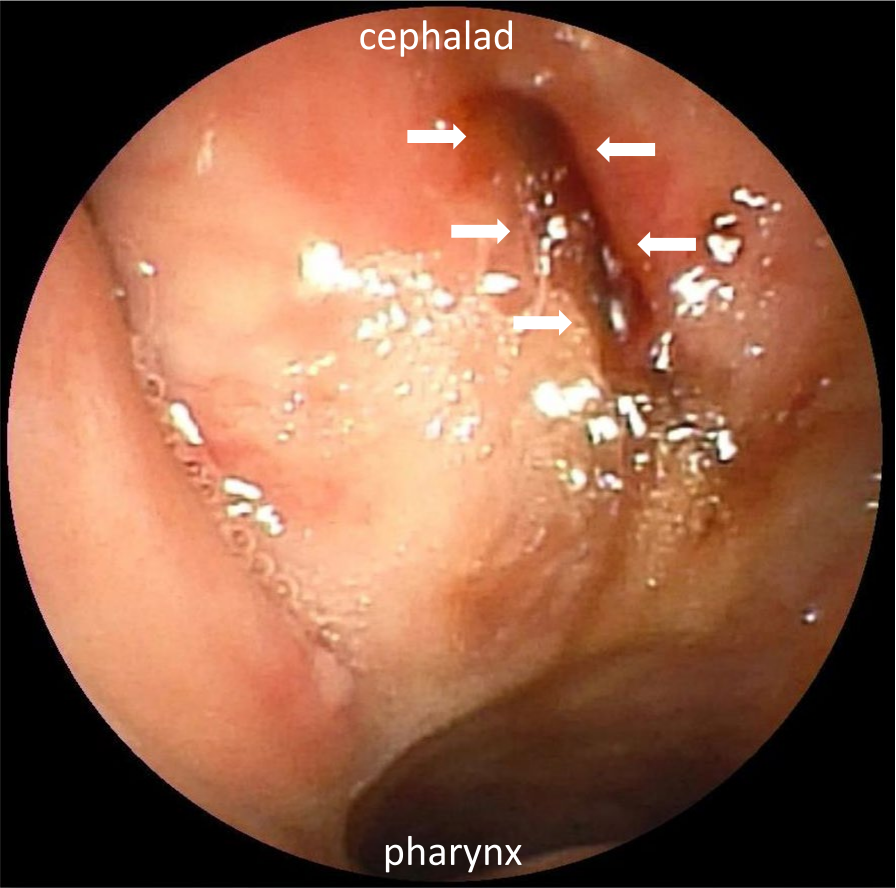
A fiber-optic image of the nasopharynx taken by nasal endoscopy. A fresh laceration of the posterior pharyngeal mucosa was observed

Chest X-ray imaging performed immediately after surgery revealed extensive subcutaneous emphysema on the right side of the neck (Fig. 1A). Contrast-enhanced computed tomography (CT) performed after extubation revealed that the retropharyngeal dissection extended to the right common carotid artery; however, contrast leakage was not observed in the region (Fig. 1B and C). Fiber-optic laryngoscopic examination of the nasal cavity and nasopharynx showed a fresh laceration of the posterior pharyngeal mucosa suggesting entry into the false passage by the nasotracheal intubation (Fig. 2). Treatment was initiated with 1-g cefazolin administered intravenously every 8 h for 5 days, followed by 500 mg of levofloxacin administered orally every 24 h for 4 days. In addition, blowing the nose was prohibited to prevent further dissection.

The patient was transferred to the ward on postoperative day 1 and started oral intake on postoperative day 2. Follow-up fiber-optic examination performed on postoperative days 2, 3, and 10 indicated the laceration gradually reduced and disappeared. The patient was discharged on postoperative day 13 without any additional complications.

## Discussion

Retropharyngeal dissection by nasotracheal tube is a possible complication of nasotracheal intubation [3–5], although only 2% of cases have been described [6]. In our case, the tube was believed to be in the vicinity of major cervical vessels; consequently, there was a danger of serious vascular injury, such as carotid dissection or thrombosis [7, 8]. We retrospectively measured the possible position of the distal tip of the tube using CT images. Actually, it was located in the most inferior part of the submucosal air. This findings show that the retropharyngeal dissection was directly caused by the tube, and the subsequent mask ventilation had little effect on the spread of tissue damage.

The process of nasotracheal intubation starts with blind insertion of the endotracheal tube from the nostril through the nasal cavity into the nasopharynx, and a relatively narrow nasal cavity causes resistance to endotracheal tube passage. Additionally, since the posterior wall of the nasopharynx is composed of loose connective tissue, the tube can easily injure the mucosa even in the absence of excessive force. To prevent this complication, a guided technique using a bougie, suction catheter, or nasogastric tube has been suggested [9, 10]; this technique was used in our second attempt. Also, fiber-optic-guided nasotracheal intubation may be a worthy alternative [3, 5], besides several other techniques, such as neck extension [11], nasal tip lifting [12], and thermosoftening treatment of the nasotracheal tube [13]. The Parker Flex-Tip nasal endotracheal tube is reported to reduce the incidence of nasal mucosal trauma compared with a conventional tip tracheal tube [14]. It has a curved distal tip, the posterior bevel, which can glide over the mucosal surface. However, in our case, the clockwise rotation of the tube might have caused the posterior bevel to perforate the mucosa. In contrast, the conventional side beveled tube is recommended to be rotated counterclockwise when resistance is felt at the pharyngeal wall [15].

More importantly, more attention should have been given to the depth of the tube. Kim et al. [11] have previously shown that, on average, the distance between the nose and the posterior wall of the nasopharynx was 9.7 ± 0.8 cm. This value would be useful for determining the lower limit of the depth for direct visualization of the tip of the tube. It has been also reported that the length of the nares-vocal cord can be predicted using height [16]. According to the formula, the nares-vocal cord length of our patient was estimated as 15.5 cm. Considering these values, we should have confirmed the tube existence in the oropharynx at the point of the tube passing over 10–12 cm from the nostril.

If retropharyngeal dissection does occur, careful follow-up of the patient’s airway using CT images and endoscopic assessment is essential. These investigations can detect bleeding, tissue swelling, hematoma, and the severity of the deep structure injury; more so, the timing of extubation must be carefully considered. It might have been better to have pre-extubation CT images in our case considering the depth of dissection. Also, observation of the patient in the intensive care unit is recommended for the timely detection of late hematoma formation or tissue swelling. Prophylactic antibiotic treatment is also recommended to reduce the risk of infective complications [4].

Therefore, although a rare complication, dissection of the retropharyngeal tissue can occur during nasotracheal intubation, and the tube can damage major cervical vessels in the vicinity. Clinicians need to master various strategies for safe nasotracheal intubation, including verifying the depth of the tube.

## Supplementary Information

**Additional file 1.** Additional images for cover letter

## Data Availability

Not applicable
