# Supplementary material for: Accidental retropharyngeal dissection extending close to the right common carotid artery during nasotracheal intubation: a case report
Source: JA Clin Rep. 2023 Feb 28;9:11. doi: 10.1186/s40981-023-00603-1 (PMC9971412; doi:10.1186/s40981-023-00603-1)
Supplement: Supplementary file 1 — Additional file 1. Additional images for cover letter [file 40981_2023_603_MOESM1_ESM.docx]

Discussion

1. *In the first paragraph of this section, the authors discuss the risk of major cervical vessel injury caused by retropharyngeal dissection. They showed the depth of the tube was 17 cm in the case presentation. Please measure possible position of distal tip of the endotracheal tube in the first attempt using their UT images to show how dangerous this present case was.*

Following the reviewer’s comment, we have added the following sentence: “We retrospectively measured the possible position of the distal tip of the tube using CT images. Actually, it was located in the most inferior part of the submucosal air. This fact shows that the retropharyngeal dissection was directly caused by the tube, and the subsequent mask ventilation had little effect on the spread of tissue damage.” In addition, we have attached CT images showing the actual measurement.
